# Diagnosis, Pathological Findings, and Clinical Management of Gangliocytic Paraganglioma: A Systematic Review

**DOI:** 10.3389/fonc.2018.00291

**Published:** 2018-07-27

**Authors:** Yoichiro Okubo, Emi Yoshioka, Masaki Suzuki, Kota Washimi, Kae Kawachi, Yoichi Kameda, Tomoyuki Yokose

**Affiliations:** Department of Pathology, Kanagawa Cancer Center, Kanagawa, Japan

**Keywords:** gangliocytic paraganglioma, literature survey, metastasis, neuroendocrine tumor, pancreatic polypeptide, progesterone receptor

## Abstract

**Background:** Although gangliocytic paraganglioma (GP) is considered a rare benign neuroendocrine tumor, cases of mortality have been reported. Occasionally, GP is misdiagnosed as neuroendocrine tumor G1, which is associated with a poorer prognosis than GP. To avoid such misdiagnoses, it is important to understand the clinicopathological characteristics of GP. Thus, herein, we discuss the current literature on the clinicopathological characteristics of GP.

**Methods:** We conducted a systematic review in accordance with the Preferred Reporting Items for Systematic Reviews and Meta-Analyses statement. PubMed and Japana Centra Revuo Medicina searches were used to identify papers describing GP. Inclusion criteria included confirmation of epithelioid, spindle-shaped, and ganglion-like cells in the main article and/or figures and whether the paper was cited in other studies of GP. Data were collected on age, sex, site of the primary lesion, tumor size, treatment, prognosis, lymph node metastasis (LNM), depth of tumor invasion, rate of preoperative diagnosis, and clinical symptoms.

**Results:** In total, 162 papers containing 263 cases of GP met the criteria. The mean age at diagnosis was 53.5 years. The male-to-female ratio was 157:104. The mean tumor size was 25.7 mm. The predominant site of the primary tumor was the duodenum (89.7%). The most common clinical sign of GP was gastrointestinal bleeding (47.9%). Other signs and symptoms of GP included abdominal pain (44.7%), anemia (20.3%), incidental findings (12.9%), nausea (6.9%), weight loss (5.5%), general fatigue (5.1%), jaundice (4.6%), and incidental autopsy findings (5.1%). LNM was observed in 11.4% of patients. Liver metastasis was observed in 1.1% of patients. Depth of tumor invasion (penetrating beyond the submucosal layer or sphincter of Oddi) was by far the most significant risk factor for LNM in patients with GP. This suggests, along with histological heterogeneity, that GP may have hamartomatous characteristics. Furthermore, immunohistochemical expression of progesterone receptor and pancreatic polypeptide were useful in distinguishing between GP and neuroendocrine tumor G1, even in small biopsy specimens.

**Conclusions:** We reveal the clinicopathological characteristics of GP, including risk factors for LNM, differential diagnostic approaches, and improvements in the clinical management of this tumor.In addition, GP may have hamartomatous characteristics.

## Introduction

### Rationale

Gangliocytic paraganglioma (GP) is considered a rare neuroendocrine tumor (NET) that typically arises in the second part of the duodenum (1). Although a few investigators have reported cases of liver metastasis (2–4) and, in one case, a fatal duodenal GP (4), this tumor is usually associated with an excellent prognosis (5). The first case report was published in 1957 (6). Two hundred and sixty-three cases have been reported to date. It is noteworthy that in our previous literature survey in 2011, we identified 192 cases of GP (7). Therefore, 71 new cases have been reported in the last seven years, suggesting that, more recently, GP may be attracting more attention from oncologists. However, GP is often misdiagnosed as NET G1 (8), despite their prognostic differences (GP is associated with a better prognosis) (9). In addition, few studies have reported on the epidemiological and clinicopathological characteristics of GP due to its rarity. Thus, we performed this systematic review.

### Objectives

We wish to provide up-to-date information on the above mentioned topics by conducting a systematic review to help oncologists gain better insights into the diagnosis and clinical management of this rare NET.

### Research question

We aimed to determine more detailed clinicopathological characteristics of GP, including risk factors for lymph node metastasis (LNM), differential diagnostic approaches (e.g., immunohistochemical staining to differentiate GP from NET G1), and improvements in the clinical management of this tumor.

## Methods

### Study design

A literature survey was performed on February 24, 2018 in accordance with the Preferred Reporting Items for Systematic Reviews and Meta-Analyses statement (10).

### Participants, interventions, comparators

This systematic review involved a cumulative case series. Therefore, timing and effect measures are based on individual case reports/series.

### Systematic review protocol

The primary outcome of this systematic review was obtaining data on age, sex, site of the primary lesion, tumor size, treatment, prognosis, presence of LNM, depth of tumor invasion, rate of preoperative diagnosis, clinical symptoms, and immunohistochemical findings. Statistical analysis was conducted using the primary extracted data.

### Search strategy

The protocol was approved by the Ethics Committee of Kanagawa Cancer Center (Kanagawa, Japan) (approval no.: 27-38). Because this study was a systematic review, the requirement for informed consent was waived.

### Data sources, studies sections, and data extraction

The term “gangliocytic paraganglioma” was entered into PubMed (http://www.ncbi.nlm.nih.gov/pubmed/) and Japana Centra Revuo Medicina (a medical database for Japanese papers; http://www.jamas.or.jp/) without limiting functions. The resultant Abstracts were screened to identify papers describing GP. After screening, the full-text articles were checked to identify papers describing GP following the inclusion and exclusion criteria outlined below.

Papers were considered to be case reports/series of GP if any of the following inclusion criteria were met: (1) epithelioid, spindle-shaped, and ganglion-like cells were reported in the main article and/or figures and (2) the paper was cited in other studies of GP. Papers were excluded if they were written in any language other than English or Japanese or were review articles.

### Data analysis

We collected data on age, sex, site of the primary lesion, tumor size, treatment, prognosis, presence of LNM, depth of tumor invasion, rate of preoperative diagnosis, clinical symptoms, and immunohistochemical findings. Statistical analyses (non-parametric Mann-Whitney *U*-tests, Chi-square tests, and multivariate logistic regression analysis) were performed using Statistical Package for the Social Sciences for Windows (software version 20; IBM Corp., Armonk, NY, USA). A *P* < 0.05 was considered statistically significant.

## Results

### Provide a flow diagram of the studies retrieved for the review

**Figure 1:**
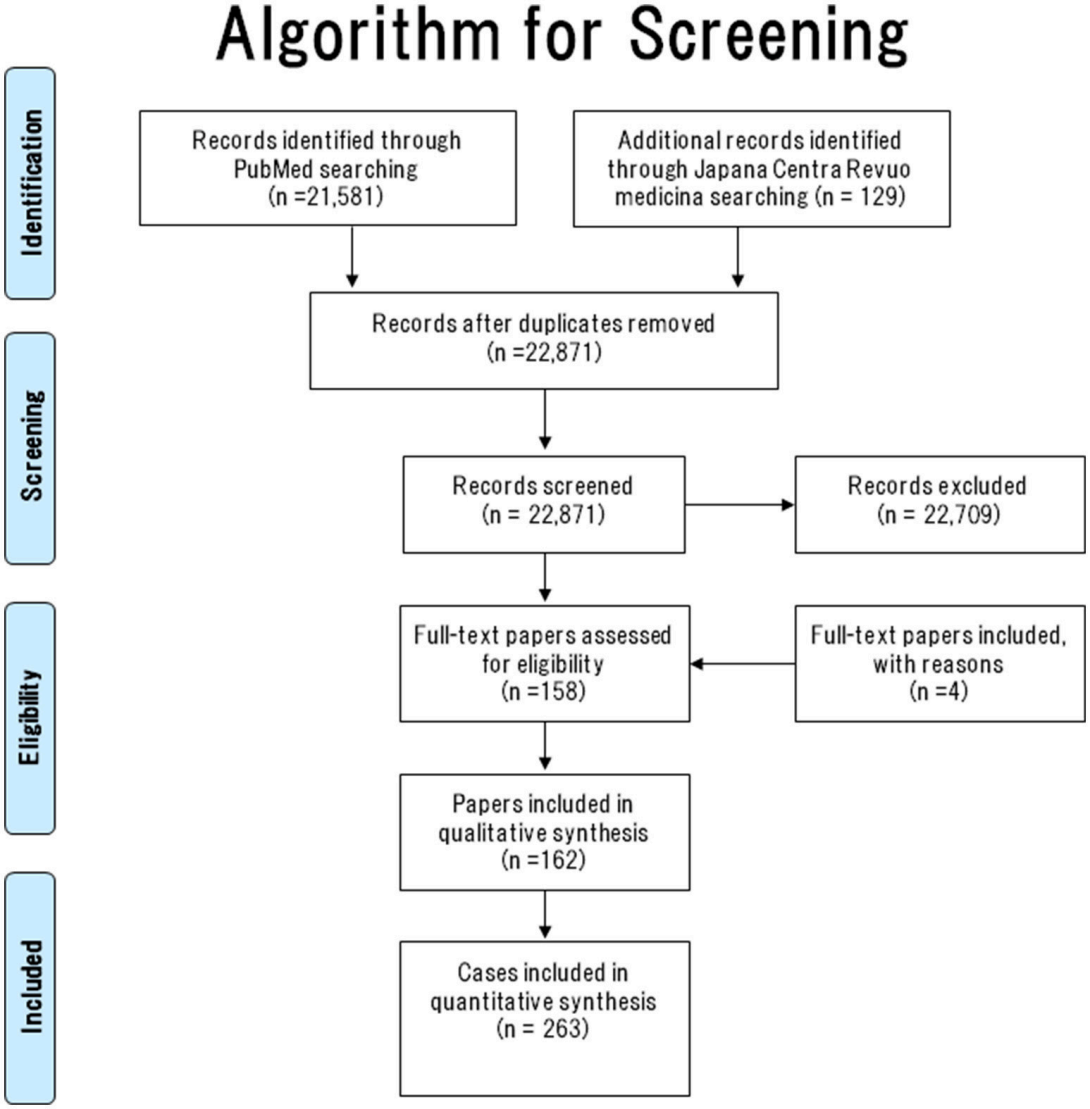
Literature search algorithm.

The literature search algorithm (flow diagram of the studies retrieved for this systematic review) is summarized in Figure 1.

### Study selection and characteristics

In total, 22,871 papers were screened (PubMed [*n* = 22,742] and Japana Centra Revuo Medicina [*n* = 129]). Following Abstract screening, 22,709 papers were excluded because they did not describe GP or were written in a language other than English or Japanese (11–29). The full-text was checked to confirm that all papers had met the inclusion criteria. In total, 158 (English [*n* = 127] and Japanese [*n* = 31]) GP case reports were identified.

Until the term “gangliocytic paraganglioma” was coined by Kepes et al. (30) in 1971, became widely known or standardized globally, GP was still being reported under other names. Therefore, we checked the main article documents, figures, and references to the 158 papers.

As a result of this search, another four papers (31–34) that met the above inclusion criteria were added. Consequently, 162 papers (158 and 4 GP case reports/series) containing 263 cases of GP were collected. The literature search algorithm (flow diagram) is summarized in Figure 1.

### Synthesized findings

#### Clinical characteristics

**Figure 2:**
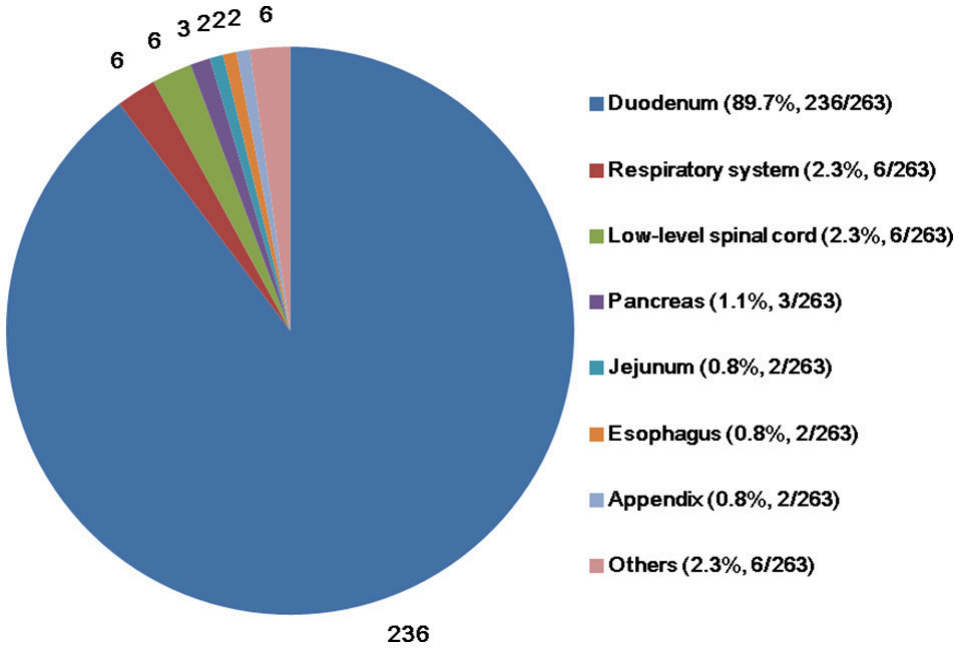
Primary sites of gangliocytic paraganglioma.

The mean age of the patients (*n* = 263) at the time of diagnosis was 53.5 (range, 15.0–84.0) years. The majority of the patients were male, with a male-to-female ratio of 157:104 (*n* = 261; two papers did not report patient sex). The mean tumor size (*n* = 203) was 25.7 (range, 5.5–100.0) mm. The predominant site of the primary tumor was the duodenum (*n* = 236; 89.7%), followed by the respiratory system (*n* = 6; 2.3%), low-level spinal cord (*n* = 6; 2.3%), pancreas (*n* = 3; 1.1%), jejunum (*n* = 2; 0.8%), esophagus (*n* = 2; 0.8%), appendix (*n* = 2; 0.8%), and other sites (Figure 2).

Clinical signs and/or symptoms were described in 217 patients. The most common clinical sign of GP was gastrointestinal bleeding (*n* = 104; 47.9%). Other signs and/or symptoms of GP included abdominal pain (*n* = 97; 44.7%), anemia (*n* = 44; 20.3%), incidental findings (*n* = 28; 12.9%), nausea (*n* = 15; 6.9%), weight loss (*n* = 12; 5.5%), general fatigue (*n* = 11; 5.1%), jaundice (*n* = 10; 4.6%), and incidental autopsy findings (*n* = 11; 5.1%).

In 263 GP cases, LNM and liver metastasis were observed in 11.4% (*n* = 30) and 1.1% (*n* = 3) of patients, respectively (2–4). GP recurrence was reported in only three patients (4, 35, 36). Unfortunately for one patient, GP was the cause of death (4). Twenty-seven patients underwent endoscopic treatment. Although one patient required additional surgery owing to the presence of residual tumor following the initial treatment (37), no additional treatments were conducted in the remaining 26 patients.

#### Pathological characteristics

**Figure 3:**
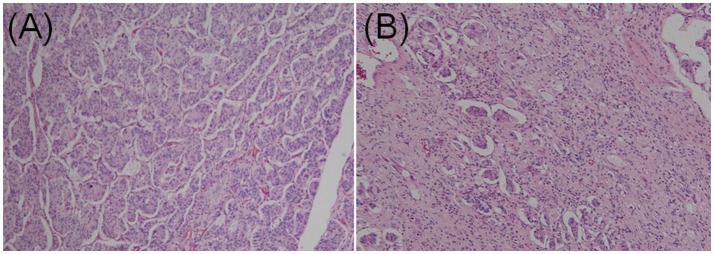
Contrasting histopathological features of gangliocytic paraganglioma. **(A)** Epithelioid cells accounted for the majority of the tumor. Dense proliferation was observed. Cells were arranged in cords in a nested Zellballen pattern. **(B)** Epithelioid cells exhibited sporadic proliferation. Spindle cells were predominant in the stroma and arranged in a chaotic pattern (hematoxylin and eosin staining; original magnification × 100).

GPs contain three characteristic cell types: epithelioid, spindle-shaped, and ganglion-like. The distribution of these three cell types often varies considerably (Figures 3A,B) (9). Each of the three characteristic cell types have unique immunohistochemical profiles (the denominator varied for each immunohistochemical marker because only papers describing positive or negative immunohistochemical findings were evaluated). The positive immunoreactivity rates of markers in the epithelioid cells were as follows: CD56 (100.0% [29/29]), synaptophysin (95.1% [97/102]), neuron-specific enolase (94.2% [98/104]), progesterone receptor (94.1% [16/17]), pancreatic polypeptide (87.1% [88/101]), somatostatin (81.0% [81/100]), chromogranin A (77.3% [119/154]), and cytokeratins (60.0% [63/105]). In spindle-shaped cells, S-100 protein exhibited the highest positive immunoreactivity rate (96.0% [167/174]), followed by neuron-specific enolase (80.5% [70/87]), neurofilament (69.3% [52/75]), CD56 (52.6% [10/19]), vimentin (50.0% [3/6]), synaptophysin (47.1% [33/70]), and chromogranin A (7.8% [9/115]). In ganglion-like cells, CD56 exhibited the highest positive immunoreactivity rate (95.2% [20/21]), followed by synaptophysin (91.3% [73/80]), neuron-specific enolase (86.0% [86/100]), somatostatin (50.6% [45/89]), chromogranin A (31.9% [36/113]), neurofilament (31.8% [23/74]), pancreatic polypeptide (31.5% [29/92]), and S-100 protein (24.8% [33/133]; Table 1).

**Table 1:** Immunohistochemical staining of gangliocytic paraganglioma.

**Immunohistochemical staining**	**Epithelioid cells**	**Spindle-shaped cells**	**Ganglion-like cells**
Bcl-2	15.4% (2/13)	53.8% (7/15)	30.8% (4/13)
Calcitonin	20.0% (5/25)	19.0% (4/21)	19.0% (4/21)
CD34	0.0% (0/2)	33.3% (1/3)	0.0% (0/2)
CD56	100.0% (29/29)	52.6% (10/19)	95.2% (20/21)
Chromogranin A	77.3% (119/154)	7.8% (9/115)	31.9% (36/113)
Cytokeratins	60.0% (63/105)	6.2% (5/81)	7.3% (6/82)
c-Kit	0.0% (0/9)	0.0% (0/12)	11.1% (1/9)
Corticotropin	100.0% (1/1)	0.0% (0/3)	0.0% (0/3)
Estrogen receptor	23.1% (3/13)	0.0% (0/12)	0.0% (0/12)
Gastrin	5.9% (4/68)	0.0% (0/63)	0.0% (0/62)
Glucagon	5.8% (3/52)	0.0% (0/47)	2.1% (1/47)
Insulin	4.0% (2/50)	0.0% (0/45)	0.0% (0/45)
Neurofilament	20.0% (15/75)	69.3% (52/75)	31.1% (23/74)
NSE	94.2% (98/104)	80.5% (70/87)	86.0% (86/100)
p53	0.0% (0/14)	0.0% (0/13)	0.0% (0/13)
Progesterone receptor	94.1% (16/17)	0.0% (0/14)	7.1% (1/14)
Pancreatic polypeptide	87.1% (88/101)	0.0% (0/90)	31.5% (29/92)
S-100 protein	12.5% (18/144)	96.0% (167/174)	24.8% (33/133)
Serotonin	20.3% (13/64)	1.4% (1/70)	15.3% (9/59)
Somatostatin	81.0% (81/100)	7.8% (6/77)	50.6% (45/89)
Synaptophysin	95.1% (97/102)	47.1% (33/70)	91.3% (73/80)
Vimentin	33.3% (3/9)	50.0% (3/6)	16.7% (1/6)
VIP	14.3% (5/35)	15.6% (5/32)	15.6% (5/32)

*Bcl-2, B-cell lymphoma 2; CD, cluster of differentiation; NSE, neuron-specific enolase; p53, tumor protein p53; VIP, vasoactive intestinal peptide*.

**Figure 4:**
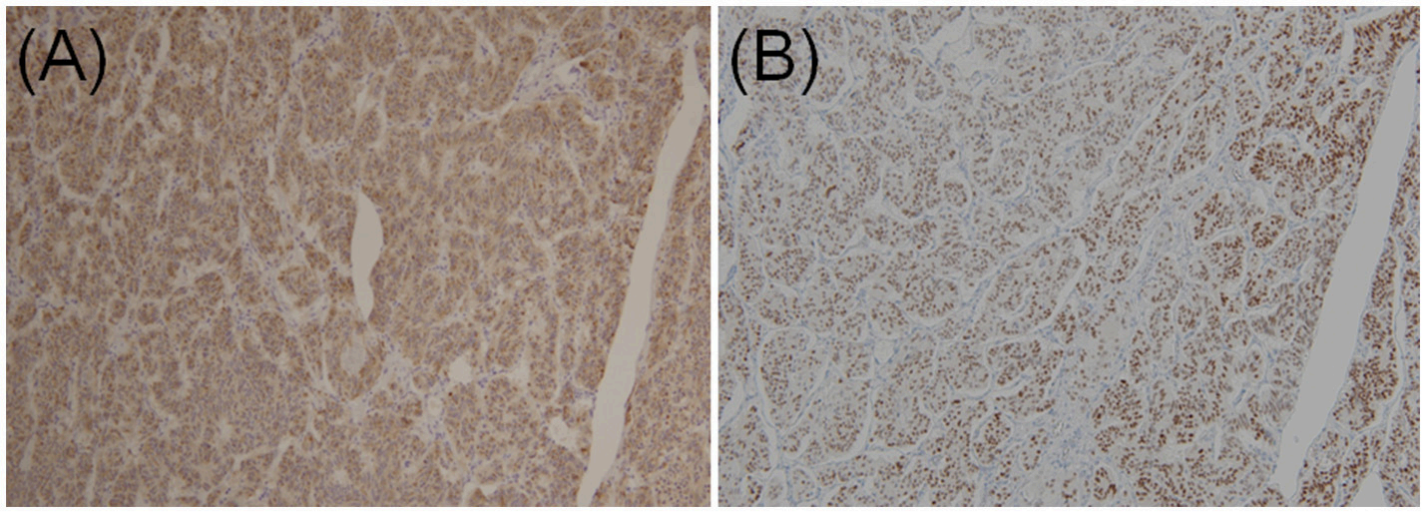
Immunohistochemical staining of **(A)** pancreatic polypeptides and **(B)** progesterone receptors in epithelioid cells of duodenal gangliocytic paraganglioma (original magnification × 100).

In two previous multi-institutional retrospective studies (9, 38), we found that the majority of GP epithelioid cells exhibited positive immunoexpression for pancreatic polypeptide and progesterone receptor (Figures 4A,B), whereas NET G1 cells stained negative for pancreatic polypeptide and progesterone receptor. Histopathological findings of biopsy specimens obtained before surgery or endoscopic treatment were described in 63 cases. However, among these 63 cases, only 12 were successfully diagnosed with GP; 41 showed no tumor cells, nine were diagnosed with or suspected of having a different NET (NET G1 [*n* = 6], paraganglioma [*n* = 2], or ganglioneuroma [*n* = 1]), and one was diagnosed with atypical cells. Thus, only 19.0% of patients were preoperatively diagnosed with GP.

#### Risk factors for LNM

In the univariate analysis, mean tumor size and depth of tumor invasion were significant risk factors for LNM in patients with duodenal GP. The mean tumor size in patients with and without LNM was 30.6 and 24.9 mm, respectively. Thus, patients with LNM had significantly larger tumors than those without LNM (Mann-Whitney *U*-test, *P* = 0.035). With respect to depth of tumor invasion (*n* = 170 cases), the rate of LNM was higher in patients with GP penetrating beyond the submucosal layer or sphincter of Oddi (*n* = 88) compared to those with GP located within these layers (*n* = 82). In the latter, six patients (7.3%) had LNM and in the former 20 patients (22.7%) had LNM (Chi-square test, *P* = 0.006; Table 2). No significant differences were observed with respect to age or sex between patients with and without LNM.

**Table 2:** Risk factors for LNM in gangliocytic paraganglioma.

**Variable**	**Patients with LNM**	**Patients without LNM**	***P*-value**
Mean tumor size (mm)	30.9	24.9	0.035^a^
Spread within the submucosal layer or sphincter of Oddi	7.3% (6/82)	92.7% (76/82)	0.006^b^
Spread beyond the submucosal layer or sphincter of Oddi	22.7% (20/88)	77.3% (68/88)	

a Mann-Whitney U-test.

b *Chi-square test*.

*LNM, lymph node metastasis*.

To elucidate the impact of these risk factors, we conducted multivariate logistic regression analysis. The results showed that both mean tumor size and depth of tumor invasion were significant risk factors for LNM. However, odds ratios differed. Mean tumor size had an odds ratio of 1.03 (95.0% confidence interval: 1.00–1.06; *P* < 0.01), while depth of tumor invasion had a higher odds ratio of 3.82 (95.0% confidence interval: 1.42–10.30; *P* < 0.01; Table 3). To further elucidate the cause of this result (given that depth of tumor invasion is an important risk factor for LNM), we conducted a statistical analysis of the relationship between mean tumor size and depth of tumor invasion. However, no significant relationship was observed. The mean tumor size in patients with tumor spread within or beyond the submucosal layer or sphincter of Oddi was 26.6 and 24.2 mm, respectively (Mann-Whitney *U*-test, *P* = 0.82).

**Table 3:** Multivariate logistic regression analysis of lymph node metastasis.

**Variable**	**OR (95.0% CI)**	***P*-value**
Mean tumor size	1.03 (1.00–1.06)	<0.001
Depth of tumor invasion	3.82 (1.42–10.30)	<0.001

*CI, confidence interval; OR, odds ratio*.

### Risk of bias

Unfortunately, because this systematic review involved a cumulative case series, there is an inherent publication bias. This is a key limitation of the data.

## Discussion

### Summary of main findings

The predominant site of the primary GP was the duodenum. There was also a slight male preponderance. Through our literature survey, we found that gastrointestinal bleeding, a cause of severe anemia, was the most commonly reported clinical symptom of GP. One patient had recurrence due to the presence of residual tumor following the initial treatment (35). These findings suggest that surgery or endoscopic treatment of GP should be considered and follow-up alone should be avoided.

Twenty-seven patients underwent endoscopic treatment, while only one patient required additional surgery owing to the margin-positive results of the initial endoscopic procedure (37). Thus, endoscopic treatment can potentially produce favorable outcomes. Indeed, two patients also received irradiation (4, 39). However, it is possible that patients with negative surgical margins may not require irradiation because no recurrence or metastasis has been reported in such patients.

It should be noted that the distribution of the three characteristic tumor cells varied considerably. To accurately diagnose GP, pathologists should be aware that the histopathological features of GP are variable.

In the present study, approximately 10.0% of patients had LNM. Univariate analysis identified mean tumor size and depth of tumor invasion as significant risk factors for LNM in patients with GP. However, multivariate logistic regression analysis showed that depth of tumor invasion was a more significant risk factor than mean tumor size. Although mean tumor size is considered an important predictive factor of tumor progression, no significant relationship was observed between mean tumor size and depth of tumor invasion in duodenal GP. Moreover, the primary layer alone influences the probability of LNM in GP (GP arising from deep layers has a higher risk of LNM). This suggests, along with histological heterogeneity, that GP may have hamartomatous characteristics.

Further discussion is warranted regarding the proliferative activity of GP. Previous studies (4, 9) have shown that GP has no significant mitotic figures or Ki-67 protein, tumor protein p53, and B-cell lymphoma two immunoexpression, which are prognostic factors for several types of NETs (40–42), irrespective of the presence of LNM. Thus, general immunohistochemical prognostic factors in NETs are not useful for assessing the malignant potential of GP tumors.

Unfortunately, a typical biopsy has limited accuracy in diagnosing GP. However, one patient was successfully diagnosed with GP following multiple boring biopsies (43). These findings suggest that a boring biopsy may be a useful adjunctive procedure in selected cases. Schwannoma, gastrointestinal stromal tumor, leiomyoma, paraganglioma, and NET G1 are examples of differential diagnoses that can be derived from biopsy specimens (5). In the majority of cases, schwannoma, gastrointestinal stromal tumor, and leiomyoma can be easily distinguished using immunohistochemical staining (S-100 protein, c-Kit, CD34, synaptophysin, and chromogranin A). Paraganglioma has similar morphological, immunohistochemical, and genetic features to GP (hypoxia-inducible factor-2α gain-of-function mutations have been detected in both GP and paraganglioma) (44, 45). Thus, pathologists should consider the primary lesion and the presence of ganglion-like cells. For instance, ganglion-like cells are absent from paraganglioma, which rarely arises from the duodenum (46). Although NET G1 is the most important differential diagnosis, GP is often misdiagnosed as NET G1 (8). Nevertheless, as a benign course is more common in cases of GP than in cases of NET G1 (47), it is important to distinguish between GP and NET G1. In previous multi-institutional retrospective studies (9, 38), we showed that immunohistochemical examination of pancreatic polypeptide and progesterone receptor could be used to distinguish between GP and NET G1, even with small biopsy specimens. Finally, our previous multi-institutional retrospective studies suggest that GP accounts for a consistent proportion of duodenal NETs.

### Limitations

Unfortunately, because this systematic review involved a cumulative case series, there is an inherent publication bias. This is a key limitation of the data.

## Conclusions

The present review of 263 cases revealed more detailed clinicopathological characteristics of GP, including risk factors for LNM, differential diagnostic approaches, and improvements in the clinical management of this rare NET. In particular, GP may have hamartomatous characteristics. Occasionally GP is misdiagnosed as NET G1. To avoid such misdiagnoses, we emphasized the utility of immunohistochemical staining with progesterone receptor and pancreatic polypeptide to differentiate GP from NET G1, even with small biopsy specimens.

## Author contributions

YO collected the data, performed the statistical analysis, and wrote the manuscript. EY, MS, and KW performed the statistical analysis and revised the manuscript. KK and YK advised the first author on GPs as senior doctors and revised the manuscript. TY performed the statistical analysis and revised the manuscript as the senior author. All authors have read and approved the final version of the manuscript.

### Conflict of interest statement

The authors declare that the research was conducted in the absence of any commercial or financial relationships that could be construed as a potential conflict of interest.

## Abbreviations

GP: gangliocytic paraganglioma
LNM: lymph node metastasis
NET: neuroendocrine tumor.
